# Herpes Zoster Oticus: Systematic Review of Clinical Prognostic Factors

**DOI:** 10.1055/s-0045-1810000

**Published:** 2026-03-11

**Authors:** Arthur Justi Cassettari, Vagner Antônio Rodrigues Silva, Agrício Nubiato Crespo

**Affiliations:** 1 Department of Otolaryngology, Head and Neck Surgery, Faculty of Medical Sciences, Universidade Estadual de Campinas (UNICAMP), Campinas, SP, Brazil

**Keywords:** facial paralysis, Herpes Zoster Oticus, prognosis

## Abstract

**Introduction:**

Herpes Zoster Oticus is etiologically associated with the reactivation of the Varicella-Zoster virus in the geniculate ganglion. Its primary manifestation includes pain, vesicles in the external auditory canal, and peripheral facial paralysis. The prognosis of the syndrome remains under scrutiny and debate, fluctuating based on symptom severity and clinical manifestation. The scarcity of articles accurately correlating clinical presentation and prognosis, coupled with individual factors and the syndrome's rarity, constrains more comprehensive analyses.

**Objectives:**

To conduct a systematic review to determine whether it is possible to predict the prognosis of RHS based on clinical factors.

**Data synthesis:**

This study identified a total of 6,464 articles within the databases, which were subsequently evaluated by two independent researchers. Sixty-two articles underwent full-text examinations, and following meticulous selection criteria, twelve articles were ultimately incorporated into this study. Factors such as age, onset of House-Brackmann degree, dizziness, hearing loss, time to treatment initiation, and comorbidities appear to exert influence on prognosis. The articles exhibit methodological limitations and discrepancies among them, stemming from variations in prognostic concepts, treatment protocols, evaluation timing, or reported outcomes. These variations hinder the possibility of conducting a methodologically sound comparison, regardless of how closely aligned the trials are with the theme.

**Conclusion:**

The review concludes that it is not possible to definitively assert the presence of a clinical parameter defining the prognosis in Herpes Zoster Oticus.

## Introduction

Peripheral facial paralysis (PFP) is the most prevalent pathology of cranial nerves, with an incidence ranging from 20 to 30 cases per 100,000 people. 1 2 3 4 Neurologist Charles Bell first described the condition in 1821 as a sudden onset hemifacial paresis. Since then, the term Bell's Palsy (BP) has been used for monosymptomatic paresis of the VII nerve without another etiological diagnosis. 5 Global literature indicates that BP remains the most prevalent form of PFP, ranging from 38 to 66% in the largest samples published to date. 6 7 Varicella-Zoster Virus (VZV) infection serves as the main differential diagnosis for BP, with a lower prevalence ranging from 4 to 12% of cases. 8 9

Initially described in 1907 by James Ramsay Hunt as a triad of symptoms consisting of pain, vesicles in the external auditory canal, and PFP, this condition is known as Herpes Zoster Oticus (HZO) or Ramsay Hunt Syndrome (RHS). Its etiology involves the reactivation of VZV in the geniculate ganglion, leading to inflammation, edema, and compression of the VII cranial nerve. The onset of PFP accompanied by ipsilateral otalgia without skin eruption can also be caused by VZV, a condition known as Zoster Sine Herpete (ZSH). 10 11 12 In addition to the classic triad of symptoms, the involvement of multiple cranial nerves may result in various clinical presentations. The most common are hearing loss, dizziness, and tinnitus due to contiguous involvement of the VIII cranial nerve and, more rarely, symptoms related to speech or swallowing, associated with the involvement of the V, IX, X, XI, and XII cranial nerves. 13

The diagnosis is typically based on the classical triad of symptoms and clinical examinations. However, it is known that the onset of vesicles can precede PFP and may be absent at the time of diagnosis. Furthermore, lesions can appear later, complicating the initial analysis and leading to confusion with BP. Although vesicles are found mostly in the external auditory canal, they may appear in other locations, particularly in the oral cavity. 14

The treatment of RSH involves a combination of antiviral agents and systemic corticosteroids, initiated as soon as possible, ideally within 72 hours of symptom onset. 15 16 Intratympanic corticosteroid therapy can also be considered. A recent meta-analysis demonstrated efficacy when applied daily in conjunction with systemic corticosteroid therapy; however, evidence remains limited, and the method requires further study. 17

The prognosis remains a subject of ongoing study and controversy, varying according to each patient and the severity of symptoms, only about 20% of patients achieve complete recovery in untreated cases. Even with appropriate treatment, the prognosis tends to be worse than that of BP. 7 Early medical treatment and appropriate therapy provide better chances of complete or nearly complete recovery. 15 Few articles accurately correlate clinical presentation with prognosis, as the rarity of the syndrome combined with a wide range of individual factors makes in-depth analysis challenging.

Currently cited prognostic factors include age, treatment onset timing, initial degree of paralysis, symptoms suggesting the involvement of other cranial nerves (such as dizziness, tinnitus, and dysphagia), adherence to treatment, and the presence of associated comorbidities, primarily Diabetes Mellitus (DM) and Systemic Arterial Hypertension (SAH). 18 19 These factors appear to influence prognosis; however, the literature diverges in attempting to establish which factors and parameters are truly fundamental in defining prognosis. Given this divergence, the objective of the present study is to conduct a systematic review to determine whether it is possible to predict the prognosis of RHS based on clinical factors.

## Methods

The review adhered to the Preferred Reporting Items for Systematic Review and Meta-Analysis Protocols (PRISMA-P) 20 guidelines and utilized the largest literature databases as research substrates: BVS – BIREME, COCHRANE, EBSCOHOST, EMBASE, PROQUEST, PUBMED, SCOPUS and WEB OF SCIENCE.

The search strategy utilized the following terms: "Herpes Zoster Oticus" OR "Zoster Sine Herpete" OR "Hunt's syndrome [Supplementary Concept]" OR "Ramsay Hunt Syndrome type 2" OR "Ramsay Hunt" OR "Ramsay AND Hunt") AND ("Facial Nerve" OR "Facial Paralysis". The initial search in the databases was conducted on July 2, 2020, and the final search was conducted on March 22, 2024.

The inclusion criteria were prospective or retrospective clinical trials related to the clinical presentation, treatment, and prognosis of PFP in cases of HZO, either through the classic presentation of SRH or ZSH.

The exclusion criteria were categorized into four groups: first, language, which ruled out studies not written in English; second, design, excluding non-clinical trial studies such as case reports, guidelines, symposiums, conference proceedings, reviews, or systematic reviews; third, classification, eliminating studies that did not utilize the House-Brackmann (HB) scale for classifying peripheral facial paralysis; and fourth, theme, where articles were excluded if their etiological diagnosis of PFP did not align with RHS or ZSH, or if they focused on patients with concomitant conditions like tumors, prior traumatic injuries, otological infections, cerebrovascular lesions, or a history of recurrent facial paralysis. Additionally, articles that did not concentrate on prognostic evaluation or that lacked this evaluation were also excluded.

The first stage involved selecting articles by evaluating titles and abstracts by two independent assessors. Conflicts were resolved by a third experienced assessor. Integration of articles from the databases was done using the Rayyan® system, available at https://rayyan.ai . Eligibility was determined through the full-text reading of selected articles by the same assessors. After the complete reading of the articles, the selected ones were included, and their data were integrated, summarized, and reviewed within this text.

## Results

A total of 6,464 articles were found in the databases. Immediately, 3,454 duplicates were excluded. The remaining 3,010 articles were selected. After the initial analysis of titles and abstracts, 62 articles were selected for full-text reading. Following the full-text reading, 50 studies were excluded, resulting in 12 articles included in this review ( Flowchart 1 ).

**Flowchart 1:**
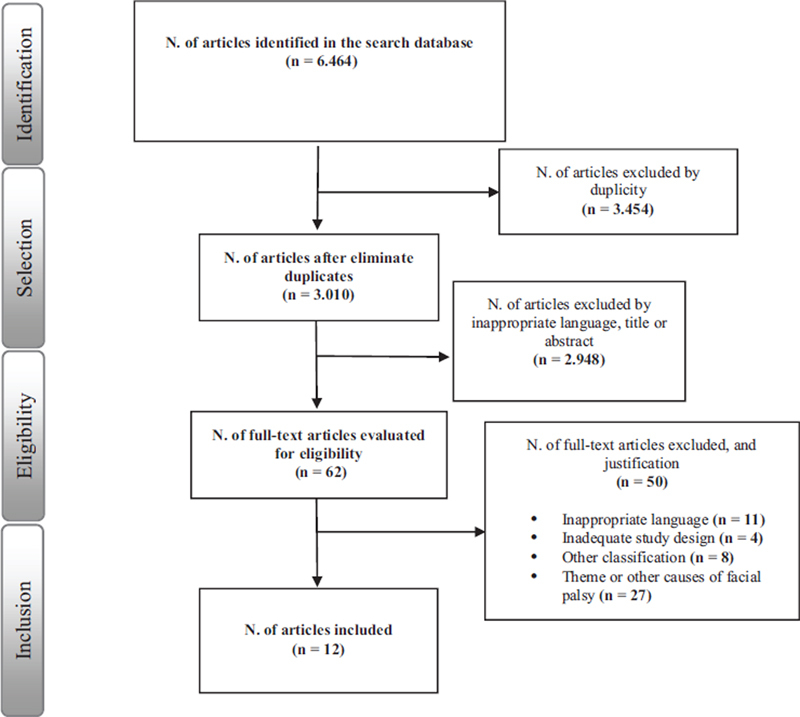
Procedure for articles inclusion in the review.

The compilation of authors, year of publication, evaluation time, and clinical factors cited as prognostic can be seen in Table 1 . Epidemiological data and treatment protocols used by each study are summarized in Table 2 .

**Table 1:** Description of the authors, year of publication, their respective samples, follow- up time and conclusions regarding the prognosis

Author (year)	VZV patients	Follow-up	Clinical Prognostic Factors (p < 0.05)
** Ko et al. (2000) 21**	n = 45	58.7 days (average)	Age, polyneuropathy*, onset degree **
** Uri et al. (2003) 22**	n = 31	12 months	Dizziness, hearing loss, treatment onset timing***
** Yeo et al. (2006) 23**	n = 26	6 months	Age, onset degree, SAH, DM, dizziness
** Kim et al. (2010) 13**	n = 11	11.3 months (average)	None
** Coulson et al. (2011) 24**	n = 101	12 months	Onset degree, incomplete eye closure, dry eye, steroid treatment after 5 days
** Ryu et al. (2012) 25**	n = 155	3 months	Onset degree, DM, SAH, age, treatment onset timing
** Byun et al. (2013) 26**	n = 22	12 months	Dizziness, hearing loss
** Shin et al. (2015) 27**	n = 81	Not included	Dizziness
** Cai et al. (2017) 28**	n = 23	6 months	Age, SAH, DM
** Kim et al. (2019) 29**	n = 106	6 months	Onset degree V or VI without comorbidities
** Kanerva et al. (2020) 14**	n = 120	6.6 years (median)	Onset degree***, treatment onset timing***
** Rim et al (2023) 30**	n = 192	12 months	None

Abbreviations: DM, Diabetes Mellitus; SAH, Systemic Arterial Hypertension; VZV, patients identified as positive for the Varicella-Zoster virus in each study.

*Polyneuropathy – involvement of any cranial nerve other than the VII nerve.

**Higher initial grade is associated with a more favorable prognosis.

***Cited by the authors as factors, without disclosing statistics.

**Table 2:** Description of authors, year of publication, epidemiological data, and most utilized treatment protocol in the sample

Authors (year)	Male: Female	Age (average)	Treatment*
** Ko et al. (2000) 21**	20:25	17–77 (45.3)	Acyclovir IV 1,5g/d 5–13 days Prednisolone O 60mg/d 7 days + reduction
** Uri et al. (2003) 22**	12:19	6–77 (50.8)	Acyclovir IV 5mg/kg 3t/day 7 days Hydrocortisone IV 300mg/d 7 days
** Yeo et al. (2006) 23**	16:10	(41.4) SD 19.4	Acyclovir IV 5mg/kg 3t/d 5 days + Famciclovir O 1,5g/d 7 days Prednisolone O 1mg/kg/d 5 days + reduction
** Kim et al. (2010) 13**	7:4	19–76 (51.5)	Acyclovir IV 750mg - 4,8g/d 5–12 days Prednisolone O 40-60mg + reduction 10–37 days
** Coulson et al. (2011) 24**	70:31	11–85 (49.4)	Acyclovir O 1g/d or Famciclovir O 750mg/d 21 days Prednisone O 1mg/kg/d 14 days + reduction
** Ryu et al. (2012) 25**	70:85	(47.2) SD 16.5	Acyclovir O 2,4g/d 5 days Methylprednisolone O 80mg/d 4 days + reduction
** Byun et al. (2013) 26**	12:10	11–82 (55)	Acyclovir or Famciclovir O 7 days** Corticosteroid*** O 1mg/kg/d 7 days + 4 days reduction
** Shin et al. (2015) 27**	42:39	19–89 (47.7)	Not included
** Cai et al. (2017) 28**	12:11	(54.43) SD 15.28	Acyclovir O 600mg/d 7-10 days Prednisone O 30mg/d 5 days + 5 days reduction
** Kim et al. (2019) 29**	40:66	(44.98) SD 16.37	Famciclovir O 750mg/d 7 days Corticosteroid** O 80mg/d 4 days + reduction
** Kanerva et al. (2020) 14**	49:71	6.9–94.7	Valaciclovir O 3g/d 7 days**** Prednisolone 60mg/d 5 days + 5 days reduction
** Rim et al. (2023) 30**	103:89	(51.3–43.2) SD 19.2–13.1	Prednisolone O 1mg/kg/d + reduction Famciclovir O 750mg/d 7 days

Abbreviations: IV, intravenous; O, orally; SD, standard deviation.

*The prevailing treatment, administered to most of the sample.

**The dosage is not specified.

***The specific corticosteroid is not specified.

****The study identified over ten treatment protocols.

Ko et al., 21 in a retrospective study published in 2000, aimed to identify prognostic factors in 45 patients with HZO. Only 17 patients underwent electroneurography. It was defined as a good prognosis of reaching HB grades I or II within 30 days of follow-up. Three variables showed statistical significance as prognostic factors: age, involvement of multiple nerves, and initial HB grade. Concerning age, the estimated improvement decreased by 0.04 points in the regression coefficient (R2) for each year of life (R2 = 2.39–0.04 x age). The involvement of multiple nerves was associated with a worse prognosis, with an impact of 0.79 on the R2. Regarding the initial grade, higher HB grades were associated with a better prognosis compared to lower grades. The study noted that patients with initial grades V and VI showed greater improvement than those with grades III and IV, potentially leading to similar outcomes with appropriate treatment, with an R2 of 0.37.

In 2003, Uri et al. 22 conducted a study to assess the response to Acyclovir treatment and prognosis in patients with RHS. The study considered a good recovery when patients reached HB grades I or II. The results showed that 65% of patients achieved complete recovery, and 82.6% were classified as having a favorable prognosis. The study also found that patients with vestibular symptoms (p = 0.029) and auditory symptoms (p = 0.05) had a worse prognosis compared to patients without these symptoms. While the analysis did not show differences in recovery rates based on age, gender, or whether treatment was initiated before or after 3 to 7 days, it did indicate better recovery rates when combined treatment was administered earlier, although a specific timeframe was not defined, and statistical significance was not observed.

Yeo et al. 23 conducted a retrospective study in 2006 aiming to evaluate prognostic factors of BP and HZO through medical record analysis. Recovery was considered when the final HB grade was lower than the initial, and complete recovery was achieved when reaching HB grade I. The study found complete recovery in 50% of HZO patients, with 84.6% considered satisfactory when reaching at least grade II of HB. Statistical significance was observed when subdividing patients by age group, showing increasing age associated with initial grade (p = 0.046), final grade (p = 0.02), and the possibility of complete recovery (p = 0.025) in HZO patients. However, there was no statistical significance in the regression analysis between age and complete recovery (p = 1.00). A worse prognosis was also noted in patients with DM, reflected in the final grade (p = 0.012) and complete recovery (p = 0.018). Patients with SAH also showed a lower degree of recovery (p = 0.025). Regarding symptoms related to the VIII nerve, patients with vertigo had a lower chance of recovery (p = 0.005).

In 2010, Kim et al. 13 conducted a retrospective review of clinical data to assess whether the prognosis of HZO presenting as polyneuropathy is worse, correlating their findings with existing literature data. The treatment was not standardized. Improvement was considered when patients reached grades I or II on the HB scale, and the PFP recovery rate was 81.8%, with 45.5% achieving complete recovery. The study highlighted that besides VII cranial nerves, other nerves commonly affected were the VIII, IX, X, V, and, more rarely, III and XII. Most of these nerves fully recovered, except for the VIII nerve. Sensorineural hearing loss was observed in nine out of eleven patients (90.9%), with complete recovery seen in only one patient (11.1%) despite combined therapy.

With a substantial caseload compared to previous literature, covering 101 RHS patients, Coulson et al. 24 in 2011 sought to independently assess prognostic factors in a retrospective cohort spanning 20 years. The study found no individual correlation between symptoms or onset pattern and recovery. The main and most significant factor influencing the outcome was the initial HB grade (p = 0.03). On average, patients improved by 3 grades on the scale, meaning those who presented with more severe paresis (grades V and VI) typically progressed to grades II or III, while patients with milder paresis (grades III and IV) achieved better recovery, reaching grades I or II, which was considered a good prognosis by the authors. Two symptoms were also associated with less improvement: incomplete eyelid closure and xerophthalmia (p < 0.01). Treatment with a single medication versus no treatment did not correlate with a better prognosis (p = 0.93); however, combined treatment had better results (p = 0.019). The study suggested that corticosteroid treatment initiated after the 5th day of onset had a better prognosis than starting earlier (p = 0.005), independent of antiviral treatment (p = 0.62). Lastly, diplopia and dysphagia indicated a tendency toward a worse prognosis, but due to the low number of patients, the authors considered statistical analysis prohibitive. They suggested that the possibility of these symptoms being correlated with diffuse polyneuropathy involving the central nervous system by VZV, diverting the diagnosis to a systemic herpetic condition.

Ryu et al. 25 conducted a prospective study in 2012 to compare patients with BP and RHS, separated by clinical presentation and treated similarly, to evaluate their respective prognostic factors after three months. The prognosis was considered good when the patient reached HB grades I or II. A higher initial HB grade correlated with a worse outcome (p < 0.05). The time to start treatment within a week also proved significant for improvement rate (p < 0.05). In all age groups, the prognosis of RHS was worse than BP (p < 0.05). RHS patients aged over 60 had a lower recovery rate than younger patients (p < 0.05). The presence of DM or SAH also indicated a worse prognosis (p < 0.05), and the combination of both conditions showed even worse outcomes compared to having only one of them (p < 0.05).

In 2013, Byun et al. 26 conducted a prospective study to evaluate the prognostic between BP and RHS patients. Only patients with HB grade IV or higher were included. Eighteen out of 22 RHS patients met the study's criteria for good prognosis. Gender, days to treatment initiation, and initial stage of PFP did not show statistical significance in predicting recovery. The presence of vestibulocochlear symptoms (dizziness and/or hearing loss) emerged as an independent risk factor for a worse prognosis, despite being present in only 6 out of 22 patients (p = 0.009). Although the study identified age over 65 as a poor prognostic factor in BP cases (p = 0.014), this was not found to be significant in RHS cases.

In 2015, Shin et al. 27 investigated the clinical manifestations of HZO, with a particular focus on dysfunctions of the VII and VIII cranial nerves and their correlation with electroneurography values. The study did not include treatment data. It found no statistical significance in the correlation between the presence or absence of hearing loss and electroneurography values. However, there was statistical significance in the group of patients with vertigo (p = 0.024), regardless of whether hearing loss was present. This finding suggests that vertigo, as a symptom, is a significant indicator of the prognosis in RHS while hearing loss alone does not significantly impact the electroneurography values in determining the outcome.

Similarly to Byun et al.'s study, 26 Cai et al. 28 in 2016 aimed to prospectively compare electroneurography results with clinical data and outcomes after six months, with a similar sample size. The study reinforced the thesis that recovery of patients with BP was superior to those with RHS after 6 months (p < 0.001). It also established that electroneurography is a significant prognostic factor in cases of PFP (p < 0.001). The authors identified age as an independent risk factor for a worse prognosis (p < 0.05), along with DM (p = 0.037) and SAH (p = 0.032). The study also suggested that the prognosis would be even worse if both comorbidities were present.

In a 2019 study, Kim et al. 29 retrospectively compared the clinical prognosis of BP with RHS. The follow-up period was 6 months, and favorable recovery was defined as achieving HB grades I and II. The study concluded that the prognosis for RHS is generally worse than for BP, however, statistically significant differences were only confirmed when the initial HB grade was severe (V or VI), coupled with electroneurography values below 10% (p = 0.036). For severe paralysis, favorable recovery was significantly lower in RHS than in BP only when there was hyperglycemia (p = 0.013), absence of obesity (p = 0.025), absence of dyslipidemia (p = 0.003), or absence of metabolic syndrome (p = 0.014). The recovery rate in patients with SAH and obesity tended to be lower in both groups but without statistical significance.

The 2020 study by Kanerva et al. 14 delved into assessing clinical prognostic factors among patients with HZO through a meticulous examination of long-term records and self-assessment questionnaires. A notable finding from the study was that a minority of patients (16%) exhibited vesicles at the onset of PFP; most cases had vesicles either preceding (48%) or following (36%) the onset of paralysis, with a small proportion (22%) of vesicles occurring in concealed areas, primarily within the oral cavity (12%). The study's conclusions highlighted that over 80% of patients achieved HB grades I or II when antiviral treatment was initiated within 72 hours of symptom onset. The study suggested that patients with a higher initial degree of PFP tended to have a more challenging prognosis. However, due to the study's limited sample size, this observation could not be statistically verified.

In the latest study incorporated into this review, Rim et al. 30 conducted a retrospective analysis of clinical features and prognostic outcomes in 192 patients diagnosed with RHS. These patients were further divided into groups based on the presence or absence of auditory impairments, with a minimum follow-up period of 12 months. The subgroup with hearing loss exhibited higher occurrences of tinnitus and dizziness (p < 0.05); however, there was no discernible difference in the rate of recovery between the two groups.

## Discussion

Ko 21 was the first and oldest article included in this review. Its primary limitation is the non-standardized evaluation period, averaging less than two months. Some patients were assessed for fewer than 30 days and, despite showing an unsatisfactory degree of recovery, were compared to patients with a much longer progression period. This issue, along with a small sample size and non-standardized treatment, can affect the interpretation of the data. The authors noted that age and the involvement of other cranial nerves negatively influence outcomes. They also suggested that a higher initial degree of paralysis might positively influence prognosis, which contradicts other studies indicating that a higher initial degree of paralysis is associated with a poorer prognosis. 14 23 24 25 29

Yeo 23 concluded that age, SAH, DM, dizziness, and the initial degree of PFP affect prognosis. However, their study was conducted retrospectively with a small sample size, comprising only 26 patients with HZO, in contrast to Kanerva's study. 14 This, on the other hand, was descriptive and lacked statistical analysis. It was also based on retrospectively collected data and included questionnaires administered to some patients long after their condition had developed, potentially leading to inaccuracies in data collection and recall bias. The patients were divided into small subgroups, which the authors themselves considered statistically unreliable. Additionally, treatments, including medications and dosages, were not standardized, preventing a reliable comparison of the data. Despite the lack of statistical confirmation, the study suggested that the onset degree and the time to start treatment appear to influence prognosis, with a significant difference in outcomes noted, particularly when treatment was initiated within 72 hours.

Uri 22 aimed to evaluate the effectiveness of combination treatment and concluded that patients with dizziness or hearing loss had a worse prognosis. The authors recommended initiating treatment as early as possible, observing that earlier treatment was associated with a better prognosis. They noted the absence of statistical confirmation, attributing to the fact that most patients began treatment within seven days of symptom onset. Nonetheless, they highlighted that outcomes were better for patients who started treatment earlier.

The only article in this review that deviates from the recommendation to initiate treatment as early as possible is Coulson. 24 Despite having a significant sample size and a one-year follow-up, this study is based on a retrospective cohort spanning 20 years and does not define a standardized treatment protocol for all patients. This lack of standardization could introduce biases such as confounding and selection biases, as not all patients underwent the same analyses. Furthermore, the study does not address comorbidities or underlying conditions that could influence the prognosis. The assertion that the initial degree of PFP directly influences prognosis is consistent with other articles. This study also uniquely highlights incomplete eyelid closure and xerophthalmia as prognostic factors. This conclusion may be directly related to the initial HB grade since incomplete eyelid closure is one of the fundamental criteria of the HB scale.

The correlation between clinical prognosis and electroneurography data was studied in the trials by Byun 26 and Cai, 28 which exhibited some methodological and sample similarities. In the first study, dizziness and hearing loss were identified as prognostic factors, whereas the second study, in a completely discrepant manner, found age, SAH, and DM to be indicative of a poor prognosis. The two studies employed different treatment protocols, particularly regarding corticosteroid dosages, as well as different follow-up periods, which may have influenced the results. In Byun's study, the type of corticosteroid used, and the antiviral dosage were not specified. The authors of this study attributed the higher chance of recovery, compared to the general literature, to the fact that most patients had vesicles preceding paralysis, which was supposedly a sign of a better prognosis, although this was not analyzed in comparative groups. Byun also concluded that the initial HB grade had little correlation with electroneurography values (p < 0.001), which could explain discrepancies when the HB grade is considered a prognostic factor. They identified electroneurography values as an independent prognostic factor, even though this was not the primary focus of the study. Both authors agree that electroneurography values are associated with a worse prognosis, but they are completely discordant regarding clinical factors.

Two studies aimed to compare the clinical outcomes of BP with HZO. Ryu 25 found a significantly lower recovery rate in HZO compared to BP. In contrast, Kim 29 concluded that HZO prognosis would only be worse than BP in cases of more severe initial grades and in the absence of comorbidities such as dyslipidemia, DM, or metabolic syndrome. However, Kim did not statistically compare patients with the same diagnosis to determine the prognostic influence of comorbidities. Based on the data presented, hyperglycemia and SAH seem to be associated with a worse prognosis in severe cases of HZO, but this was mentioned without statistical validation. Ryu also reported that a diagnosis at grade V or VI is definitively associated with a lower recovery rate, corroborating Kim's mention of SAH and DM. Both studies identified age and the time to start treatment (<7 days) as clinical factors correlated with prognosis. A noted limitation of Ryu's study is the follow-up period of only three months, which is shorter than in most studies and may compromise the evaluation of long-term outcomes.

Three studies attempted to correlate the involvement of other cranial nerves with prognosis. The study by Kim 13 is descriptive, merely mentioning cases and their outcomes without including a control group. The sample size is limited, and data were collected retrospectively, likely due to the rarity of polyneuropathy cases. Despite these limitations, the outcomes are consistent with those of other studies in this review and the broader literature, indicating that polyneuropathy cannot be firmly established as an isolated prognostic factor. Shin's study 27 separates groups based on the involvement of the eighth cranial nerve and identifies dizziness as a prognostic factor. However, the study's methodology is unclear; it lacks details on treatment and follow-up duration. Additionally, it focuses solely on these symptoms without isolating potential confounding factors such as epidemiological comparison groups or comorbidities, which compromises the analysis. The most recent study, Rim, 30 with the largest sample size, concludes that hearing loss is not a prognostic factor in HZO. This study shows that the auditory recovery rate was worse than in cases of idiopathic sudden deafness reported in the literature. It suggests that the hearing loss group may require adjunctive treatments such as intratympanic corticosteroid therapy, but it does not specify the criteria for this treatment. The study acknowledges several limitations, including its retrospective nature and the lack of adequate auditory evaluation using resources like evoked potentials or otoacoustic emissions. Additionally, it points out that dizziness could not be adequately analyzed due to a lack of parameters. Importantly, the study does not differentiate between patients with tinnitus or dizziness when evaluating prognosis, which limits the interpretation of outcomes for patients with eighth nerve involvement.

The studies included in this review, despite their relevance to the topic, exhibit several limitations and discrepancies, including variations in treatment protocols, evaluation timeframes, assessed parameters, and methodologies. These differences hinder an effective methodological comparison across the studies. Given that HZO is a rare condition, most prognostic evaluation clinical trials are conducted retrospectively due to the challenges of conducting a prospective study, which would be slow and labor-intensive. This retrospective approach can lead to information biases, such as inefficient data recording, notes that may lack reliability, and the inability to rectify errors. Additionally, there may be selection biases stemming from variations in treatments due to protocol changes over the years. Even among the few studies conducted prospectively, there are disparate results, further complicating efforts to draw conclusive findings. These challenges highlight the complexities inherent in studying a rare condition like HZO and underscore the need for careful consideration of study design and methodology in future research endeavors.

A major limitation of this review is the lack of standardization in control groups and treatments, as well as the independence in evaluating factors across the selected studies. This lack of uniformity hinders an effective methodological comparison among them. Some studies use BP as a control group, while others compare their results solely with the existing literature or are purely descriptive, focusing on specific criteria such as vestibulocochlear symptoms or polyneuropathy. This narrow focus overlooks potential confounding factors in their conclusions, such as comorbidities, age, or variations in treatment protocols.

The methodologies employed in these studies are also not easily comparable. Studies with shorter evaluation times often yield different conclusions from those with longer analysis periods, suggesting that premature outcome assessments may have occurred. Additionally, even articles with similar analysis times may present conflicting results, further complicating the ability to draw definitive conclusions from the collective data. The diagnostic method for HZO itself presents discrepancies among studies. In many cases, patients were categorized as BP or RHS based solely on clinical history and physical examination. Given that typical lesions may not always be synchronous, some studies may have been affected by selection bias. The absence of a practical and objective diagnostic method to differentiate between these conditions hinders greater diagnostic accuracy and could potentially interfere with the study results.

Another factor complicating the comparison between studies is the lack of clarity and standardized protocol in defining what constitutes a good prognosis. Throughout the literature, it is observed that most authors consider favorable prognosis when a patient reaches at least HB grade II, using this parameter for intergroup comparison. However, some studies define a good prognosis as complete improvement, or any improvement compared to the initial condition. It is important to note that patients with HB grade II may still experience sequelae, albeit mild, that can impact their quality of life. Therefore, the assertion that grade II represents a good prognosis may be debatable. Additionally, only about 50% of patients achieve complete resolution according to the studies reviewed, whereas approximately 80% reach grade II with appropriate treatment. If prognostic statistical analyses were conducted considering complete resolution as the benchmark, the results might have differed from those reported in the literature. This highlights the need for standardized criteria and clearer definitions of what constitutes a favorable prognosis in future studies on HZO.

This review aimed to maintain standardization by including only trials that utilized the HB scale as a classification method, given its widespread use in clinical practice and literature, despite its technical limitations. It is worth noting that the HB scale is subjective and examiner-dependent, which can potentially introduce bias and affect the study results. Few studies employ two evaluators with blinding, a method that could mitigate this issue.

Corticosteroiditerature data and supported by this review, there appears to be a consensus that the prognosis of RHS is significantly improved with appropriate treatment following current protocols, which typically involve combined systemic corticosteroid and antiviral therapy compared to no treatment. Furthermore, it is generally agreed upon that isolated corticosteroid therapy is inferior to combined treatment with antiviral medication. The timing of treatment initiation is a point of discussion in the management of RHS. Most authors emphasize the critical importance of starting treatment as early as possible, ideally within the first 72 hours of symptom onset. However, one author mentioned in this review suggests that the optimal time to begin corticosteroid therapy would be from the peak inflammation of the facial nerve, typically around the fifth day of symptom onset. 24

Regarding the evaluation of comorbidities, many studies included only the presence or absence of certain conditions, without considering clinical compensation, specific treatment types, or the time the patient has been living with those conditions. Similarly, the factor of age, which was often assessed independently in some studies and in groups or without considering pre-existing health conditions in others, may introduce selection bias, particularly given the higher likelihood of elderly having comorbidities.

Some symptoms that could potentially influence prognosis related to the eighth cranial nerve, such as hearing loss, tinnitus, and dizziness, were evaluated solely based on clinical reports in many studies. Confirmation through complementary exams was lacking in some cases, and information was sometimes gathered from medical records or later self-assessment, introducing potential memory biases. Studies that attempted to correlate these symptoms with complementary exams in a prospective manner often had limited sample sizes or failed to assess potential confounding factors such as treatment or the presence of comorbidities, which limited data analysis. Despite these limitations, dizziness emerged as the symptom most correlated with prognosis, although many studies did not demonstrate statistical significance. Interestingly, the most recent study did not identify dizziness as a prognostic factor, nor did it find hearing loss to be predictive of prognosis. However, an important observation from these studies is that the prognosis for hearing loss itself appears to be poor when present in HZO. Even with adequate treatment and some degree of recovery, a certain level of hearing impairment may persist.

Regarding polyneuropathy, one of the articles questions whether involvement beyond the eighth cranial nerve could be a diagnostic confusion with HZO. Even though the etiology in these cases is proven to be the reactivation of VZV, involvement beyond the nerves of the internal auditory canal could be related to a broader condition, involving the central nervous system, which goes beyond the basic concept of HZO and cannot be framed as a prognostic comparison. The article attempting to correlate polyneuropathy and prognosis, even with a limited sample, failed to prove a worse outcome. 13

In a single study, dry eye and tearing were considered symptoms that predispose to a worse prognosis. 24 However, this assertion may be correlated with a higher initial grade on the HB scale, as incomplete eye closure can hinder proper eye lubrication and increase the sensation of dry eye.

While not the primary focus of the study, complementary evaluation through electroneurography provides an objective parameter and appears to influence prognosis prediction. However, there is no consensus in the literature regarding the optimal timing for performing electroneurography, as well as the denervation thresholds that define a poor prognosis. Electroneurography conducted on the orbicularis muscle between 4 to 6 days after the onset of PFP and between 13 to 15 days in the nasal and upper lip elevator muscles appear to have greater prognostic value in acute PFP. 31

Despite the numerous limitations encountered in the literature, it is evident that certain clinical factors play a role in influencing the prognosis of HZO. These factors include age, the initial degree of paralysis, dizziness, time to start treatment, SAH, and DM. However, definitive factors or cutoff points among these variables cannot be firmly established. It is conceivable that a combination of these factors may contribute to an unfavorable prognosis in the progression of the syndrome.

The existing literature lacks individualized prospective analyses with significant sample sizes and well-established treatment protocols that correlate symptomatology with complementary exams and the degree of paralysis with electroneurography data to define prognostic criteria confidently. Given this gap in knowledge, exploring different treatment protocols aimed at improving recovery rates in these patients could prove beneficial. Further research with a more comprehensive approach and standardized methodologies is essential to enhance our understanding of prognostic factors and improve outcomes in individuals with HZO.

## Conclusion

Based on the data collected in this review and considering the numerous limitations mentioned, it is not possible to establish a definitive clinical parameter to predict the prognosis of Ramsay Hunt syndrome.
